# Optimized Wire Grid Modeling Method for Complex Metal Mesh Fabrics Using Waveguide-Contact Measurement

**DOI:** 10.3390/s26082445

**Published:** 2026-04-16

**Authors:** Kitae Park, Sia Lee, In-Sung Park, Chang-Won Seo, Seong-Sik Yoon, Jae-Wook Lee

**Affiliations:** 1 Department of Electronics and Information Engineering, Korea Aerospace University, Goyang 10540, Republic of Korea; ktpark1997@kau.kr; 2 Satellite System 2 Team, Hanwha Systems, Yongin 17121, Republic of Korea; siashaa@hanwha.com (S.L.); qkrdlstjd00@hanwha.com (I.-S.P.); seocw0420@hanwha.com (C.-W.S.); yss111@hanwha.com (S.-S.Y.)

**Keywords:** mesh reflector antenna, surface impedance, effective wire radius, Casey model, deployable antenna

## Abstract

**Highlights:**

**What are the main findings?**

- A procedure for estimating the effective wire radius *r_eff_* of the Casey surface impedance model per frequency band for complex woven/knitted mesh fabrics was presented, confirming that the reflection characteristics across each measured band can be accurately reproduced with a single parameter.
- Free-space reflection coefficients of three mesh specimens were derived via 162 waveguide-contact measurements (3 specimens × 3 waveguides × 2 polarizations × 9 repeats), confirming that directional *r_eff_* values differ by up to 1.78× due to the anisotropy of the mesh weave structure.

**What are the implications of the main findings?**

- The proposed scaling procedure can determine model parameters solely from reflection coefficient measurements without relying on cross-sectional measurements or manufacturing specifications of the mesh fabric, making it applicable to complex woven/knitted structures.
- The band-by-band *r_eff_* estimation results can be used to characterize reflective performance at target frequency bands and analyze polarization dependence in mesh reflector antenna design.

**Abstract:**

Metal mesh reflective surfaces are widely used in deployable antennas mounted on satellites where lightweight and stowability are required; however, quantitative characterization of reflective performance is difficult due to complex woven/knitted structures. This paper presents a modeling method that characterizes the reflection coefficient of complex mesh fabrics by combining a per-band effective wire radius *r_eff_* estimation procedure with the Casey surface impedance model. The lattice spacing is fixed from the specimen geometry, the electrical conductivity is set to the material property of gold (σ = 45.2 MS/m), and *r_eff_* is determined as a single parameter that minimizes the error against the measured reflection coefficient in each frequency band. For validation, waveguide-contact measurements were performed on three Atlas-series mesh specimens fabricated with gold-coated molybdenum wire (diameter: 30 μm), measuring each specimen across all three waveguide standards (WR-340, WR-90, WR-28) with nine repeated trials per configuration, totaling 162 measurement runs. The estimated *r_eff_* ranged from 10.1 to 44.5 μm depending on band and polarization, with RMSE below 0.021 dB in all native-band fits. Even for the same specimen, directional *r_eff_* values differed by up to 1.78× due to the anisotropy of the weave structure, confirming that polarization dependence must be considered in mesh reflector antenna design.

## 1. Introduction

As the satellite market grows rapidly under private-sector leadership, high-gain antennas have become essential even for satellites with constrained mass budgets [1]. Missions involving remote sensing, communications, and radio surveillance, where link budgets are stringent, demand antenna systems that deliver high gain while satisfying mass and stowage volume constraints. A representative approach to meeting these requirements is the deployable mesh reflector antenna, which uses a metallic mesh as the reflective surface and has a long heritage of development.

Mesh reflector antennas have been validated through numerous in-orbit missions. Representative examples include RaInCube [2,3], which implemented a Ka-band precipitation radar on a compact CubeSat platform; ETS-VIII [4], which demonstrated in-orbit deployment of a large-aperture geostationary reflector; and SMAP [5], which operated a 6 m class rotating reflector for L-band soil moisture observation. Recently, research targeting small satellite payloads has progressed across multiple groups, covering structural design, deployment mechanisms, reflective surface shape maintenance, and electrical performance verification [6,7,8,9,10,11]. Topics include shape-deformation-aware design [6], surface-shape measurement of truss-based prototypes [7], thermo-structural behavior under various knitting conditions [8], tension and shape optimization of cable-net reflective surfaces [9], large-deformation dynamics during deployment [10], and boundary-tension optimization considering elastic deformation [11]. In addition, Bettermann et al. [12] reviewed warp-knit patterns used in deployable mesh reflectors, and Ploeckl et al. [13] reported RF characterization results, including reflection and transmission measurements for metallic mesh specimens.

Satellite-borne antennas require reliable characterization of the reflective surface at the design stage, which in turn demands accurate modeling of the mesh fabric properties. Because design changes are virtually impossible after launch, modeling research on mesh fabric properties has been conducted extensively [14,15,16,17,18,19,20,21,22,23,24,25,26]. Establishing equivalent models that reduce uncertainty in reflective performance characterization remains an open problem.

Electromagnetic modeling of mesh fabrics builds on Astrakhan’s lattice-based approach [14,15,16]. Hill, Wait, and Casey et al. broadened the analysis by presenting current-based formulations that supplement averaged boundary conditions [17,18,19,20]. Their formulations handle the current distributions induced in the lattice more rigorously, incorporating junction discontinuities and mutual coupling effects. However, these models fundamentally consider only the dominant mode; therefore, as frequency increases and the lattice period becomes non-negligible compared to the wavelength, accuracy may degrade due to excitation of higher-order Floquet modes. Yatsenko et al. incorporated these multi-mode effects through higher-order impedance boundary conditions, extending the valid frequency range [21].

Attempts to apply this theoretical foundation to actual mesh fabric modeling were systematized mainly by the Rahmat-Samii research group [22] and organized into the strip aperture model and the wire grid model. Miura and Rahmat-Samii [23] extended this framework using the periodic method of moments (MoM) combined with physical optics to numerically analyze complex weave patterns, showing that cross-polarization levels vary significantly with the weave structure. However, their full-wave validation was limited to transmission loss, and reflection coefficient characterization against measurements was not addressed. The difficulty of constructing accurate 3D unit-cell geometry for complex woven/knitted fabrics further limits the practical applicability of full-wave approaches, motivating the need for measurement-based calibration methods. Because both models presuppose an ideal lattice structure, directly matching model parameters to physical dimensions is difficult for cases with complex geometries, such as actual woven/knitted mesh fabrics. To handle this mismatch, a scaling factor was introduced that applies correction factors to the geometric parameters of the model, defining effective parameters that reproduce measured results rather than relying on physical dimensions alone. Research was conducted to validate the equivalence between strip width and wire diameter through the scaling factor β [24], and attempts to apply the wire-grid model to mesh fabrics with complex knitted patterns using this β-based diameter conversion were also presented [25]. Meanwhile, Hyun et al. [26] introduced an effective wire spacing concept in the wire grid model to evaluate the shielding performance of rebar structures.

However, each of the above approaches has limitations. Reference [25] showed that reflection coefficients can be characterized by applying the Astrakhan PEC wire-grid model to complex mesh fabrics with a β-based wire diameter conversion, but the mesh is assumed to be a perfect conductor, so losses due to finite conductivity and frequency dependence are not reflected. Reference [26] demonstrated the applicability of scaling through the effective spacing concept in the wire grid model, but remained focused on transmission coefficient (shielding effectiveness) and did not extend the approach to reflection coefficient characterization. In other words, no case has been reported to date of applying a scaling procedure to a wire grid model incorporating finite electrical conductivity for the reflection coefficient characterization problem, which is central to mesh reflector antenna design. The wire grid model with the Casey formulation is particularly suitable for this purpose, as the internal wire impedance naturally incorporates finite-conductivity effects, including resistive loss and inductive reactance, which the strip aperture model does not explicitly capture. This paper presents a modeling method that combines an effective wire radius *r_eff_* estimation procedure with the Casey surface impedance model to quantitatively characterize the reflective performance. Compared to full-wave numerical methods that require detailed three-dimensional modeling of the unit cell, this semi-empirical approach offers a computationally lightweight alternative that relies only on macroscopic geometric parameters and a single measured dataset.

## 2. Mesh Modeling

### 2.1. Mesh Textile Geometry Considered in This Study

**Figure 1:**
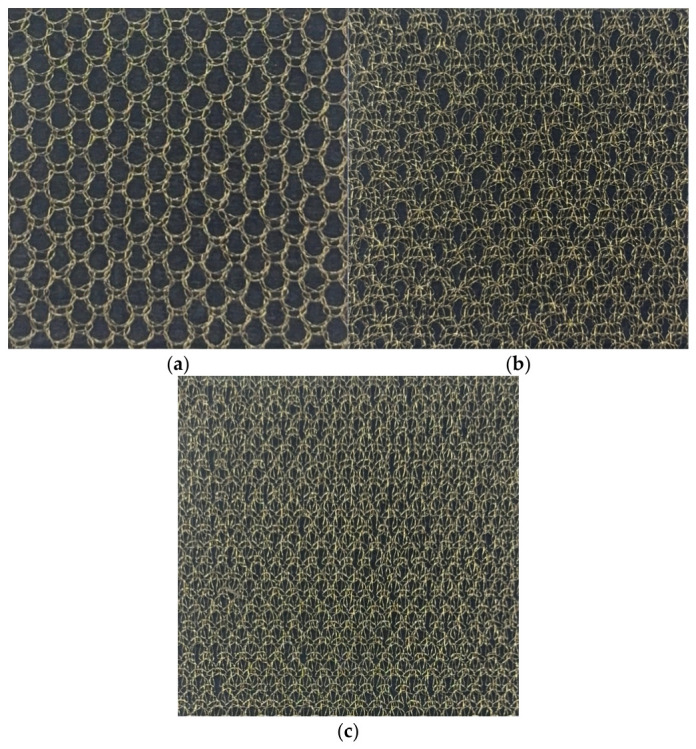
Optical images of mesh specimens fabricated by KUKDONG Telecommunication Co., Ltd. (KDT): (**a**) S-band 1in3 Atlas mesh, (**b**) X-band 2in2 Atlas mesh, and (**c**) Ka-band 1in1 Atlas mesh.

The subject considered in this paper is a metallic mesh fabric with a complex woven/knitted structure. Figure 1 shows optical images of three Atlas-series mesh specimens fabricated by KUKDONG Telecommunication Co., Ltd. (KDT) (Nonsan, Republic of Korea) using gold-coated molybdenum wire with a diameter of 30 μm (radius 15 μm): (a) S-band 1in3 Atlas (one-in-three needle pattern), (b) X-band 2in2 Atlas (two-in-two needle pattern), and (c) Ka-band 1in1 Atlas (one-in-one needle pattern). As the frequency band increases, the metallic wire is designed to be placed more densely and the aperture size to become smaller, in order to satisfy the required performance for each frequency band. The lattice period used in this paper refers to the inner spacing (wire-to-wire clearance) measured from the optical microscope images of the specimens: a_H = 1389 μm, a_V = 1900 μm for S-band 1in3 Atlas; a_H = 892 μm, a_V = 1565 μm for X-band 2in2 Atlas; and a_H = 485 μm, a_V = 1643 μm for Ka-band 1in1 Atlas. In subsequent sections, a method is presented for converting this fabric geometry into equivalent parameters of a rectangular periodic structure and characterizing reflective performance by combining the Casey surface impedance model with the scaling procedure.

### 2.2. Conventional Mesh Modeling Based on the Casey Surface-Impedance Approach

This paper uses Casey’s surface impedance-based wire mesh model [17,20] as the reference model for characterizing the reflective performance of complex woven/knitted mesh fabrics. Although the Casey model is sometimes summarized as the square-lattice case, the basic formulation includes rectangular periodic structures with different horizontal and vertical periods. This section organizes the surface impedance in a form directly usable for reflection coefficient calculation under normal incidence conditions in free space.

A rectangular wire mesh is defined by horizontal and vertical periods $a_{H}$ and $a_{V}$, where the period refers to the inner spacing (wire-to-wire clearance excluding the wire diameter). According to [20], the axial electric field on the wire surface is given as the product of the internal impedance per unit length $Z_{w}^{\prime }$ and the induced current:

(1) $$E_{H}=Z_{w}^{\prime }I_{H},\;\;E_{V}=Z_{w}^{\prime }I_{V}$$

The $Z_{w}^{\prime }$ in Equation (1) is the internal impedance per unit length of the cylindrical wire, including loss and phase delay determined by finite electrical conductivity σ. Assuming a homogeneous cylindrical conductor, the internal impedance is:

(2) $$Z_{w}^{\prime }=R_{w}^{\prime }\frac{\sqrt{j\omega \tau_{w}}I_{0}\left(\sqrt{j\omega \tau_{w}}\right)}{2I_{1}\left(\sqrt{j\omega \tau_{w}}\right)}$$

where $R_{w}^{\prime }=\frac{1}{{\sigma \pi r}^{2}}$ is the DC resistance per unit length, $\tau_{w}\;=\;{\mu_{0}\sigma r}^{2}$ is the diffusion time constant of the cylindrical conductor, and $I_{0}(\cdot )$ and $I_{1}(\cdot )$ are the zeroth- and first-order modified Bessel functions, respectively.

The surface impedance matrix $Z_{s}$ as an equivalent model averaged over a unit lattice area is defined as:

(3) $$\left[\begin{array}{c} {\tilde{E}}_{H}\\ {\tilde{E}}_{V} \end{array}\right]=\left[\begin{array}{cc} Z_{s,HH} & Z_{s,HV}\\ Z_{s,VH} & Z_{s,VV} \end{array}\right]\left[\begin{array}{c} {\tilde{J}}_{s,H}\\ {\tilde{J}}_{s,V} \end{array}\right]$$

(4) $${\tilde{J}}_{s,H}=\frac{{\tilde{I}}_{H}}{a_{V}},\;\;{\tilde{J}}_{s,V}=\frac{{\tilde{I}}_{V}}{a_{H}}$$

Under normal incidence, the in-plane wave number components $k_{x0}\;=\;k_{y0}=\;0$, so the cross-coupling terms vanish and $Z_{s,HV}\;=\;Z_{s,VH}=\;0$. Therefore, the two orthogonal components are separated into diagonal surface impedances.

Organizing Casey’s low-frequency approximation result under normal incidence and free-space conditions, the final surface impedance is:

(5) $$Z_{s}\left(\theta =0\right)=\left[\begin{array}{cc} Z_{w,V}+j\frac{\eta_{0}k_{0}a_{V}}{2\pi }L_{1V} & 0\\ 0 & Z_{w,H}+j\frac{\eta_{0}k_{0}a_{H}}{2\pi }L_{1H} \end{array}\right]$$

where $Z_{w,H}=\;Z\prime w\;\cdot \;a_{H},\;Z_{w,V}=\;Z\prime w\;\cdot \;a_{V}$, and $\eta_{0}$ and $k_{0}$ are the free-space wave impedance and wave number, respectively. $L_{1H}$ and $L_{1V}$ are the lattice-sum terms for the rectangular periodic structure and are expressed in closed form under normal incidence and free-space conditions as:

(6) $$L_{1q}=-\ln\left(1-e^{-2\pi r/q}\right),\;\;q\in \left\{a_{H},a_{V}\right\}$$

where r is the wire radius. Equations (5) and (6) maintain physical dependence on the rectangular period and wire properties while providing the response of two orthogonally polarized waves as separate equivalent surface impedances under normal incidence conditions.

### 2.3. Scaling Procedure and Effective Wire Radius Extraction

In Equations (5) and (6), the wire radius r influences both the internal impedance $Z_{w}^{\prime }$ (through $R_{w}^{\prime }$ and $\tau_{w}$ in Equation (2)) and the lattice-sum term $L_{1q}$, acting sensitively on the magnitude and phase of the predicted reflection coefficient. Since actual woven/knitted mesh fabrics have complex geometries, it is not appropriate to directly determine the geometric parameters of the cylindrical wire array model from physical dimensions. Therefore, instead of fixing the wire radius as input from manufacturing specifications or cross-sectional measurements, this paper proposes a procedure to derive the effective wire radius *r_eff_* of the model from the measured reflection coefficient. The electrical conductivity σ is fixed at that of gold, σ = 4.52 × 10^7^ S/m (45.2 MS/m).

First, the surface impedance $Z_{s}(\theta =0)$ is calculated from Equations (5) and (6), and this is used for reflection coefficient calculation at the free-space boundary. Since the wire grid acts as a shunt impedance in the free-space transmission-line model [17], the reflection coefficient under normal incidence is calculated from the equivalent surface impedances $Z_{s,HH}$, $Z_{s,VV}$ as:

(7) $$\Gamma_{H}\left(f,r\right)=\frac{-1}{1+\frac{2Z_{s,HH}\left(f,r\right)}{\eta_{0}}},\;\;\Gamma_{V}\left(f,r\right)=\frac{-1}{1+\frac{2Z_{s,VV}\left(f,r\right)}{\eta_{0}}}$$

where $Z_{s,HH}$ and $Z_{s,VV}$ are the diagonal components of Equation (5), and *r_eff_* is treated as the unknown parameter of the model.

In this study, a single effective wire radius *r_eff_* is defined for each measured frequency band and determined as the value that minimizes the error across the band between the measured reflection coefficient $\Gamma_{meas}(f)$ and the model prediction $\Gamma (f,\;r_{eff})$:

(8) $$r_{eff}\left(f\right)=\underset{r}{\mathrm{argmin}}{\left|\Gamma_{meas}\left(f\right)-\Gamma \left(f,r\right)\right|}^{\;\;\;2}$$

Here, *r_eff_* need not be identical to the physical radius of the yarn and is interpreted as a specimen-intrinsic equivalent parameter reflecting the structural features of a given mesh fabric, such as yarn geometry, knitting pattern, and inter-wire contact conditions. The per-band estimation in Equation (8) is a procedure for extracting this fabric-specific parameter within the frequency range where measurement data are available. By using a single parameter across the measured band, the reflection characteristics can be consistently reproduced without being affected by localized fluctuations at specific frequencies.

## 3. Measurements

### 3.1. Measurement Setup and Procedure

A waveguide-contact reflection coefficient measurement method was applied to characterize the reflective properties of mesh specimens. This method provides stable normal-incidence conditions for flat specimens of limited size and offers good repeatability and reproducibility.

All three Atlas-series mesh specimens in Figure 1 were measured using each of the three waveguide standards (WR-340, WR-90, WR-28), for multi-band characterization. For each specimen–waveguide–polarization combination, nine repeated trials were performed with specimen remounting between trials, yielding a total of 3 × 3 × 2 × 9 = 162 measurement runs. The measurement uncertainty was evaluated following the Guide to the Expression of Uncertainty in Measurement (GUM) [27]: the Type A standard uncertainty was obtained from the standard deviation of the nine repeated observations for each configuration, and the expanded uncertainty was reported at a 95% confidence level using the coverage factor from the t-distribution. The waveguide standards and native measurement bands are summarized in Table 1.

**Figure 2:**
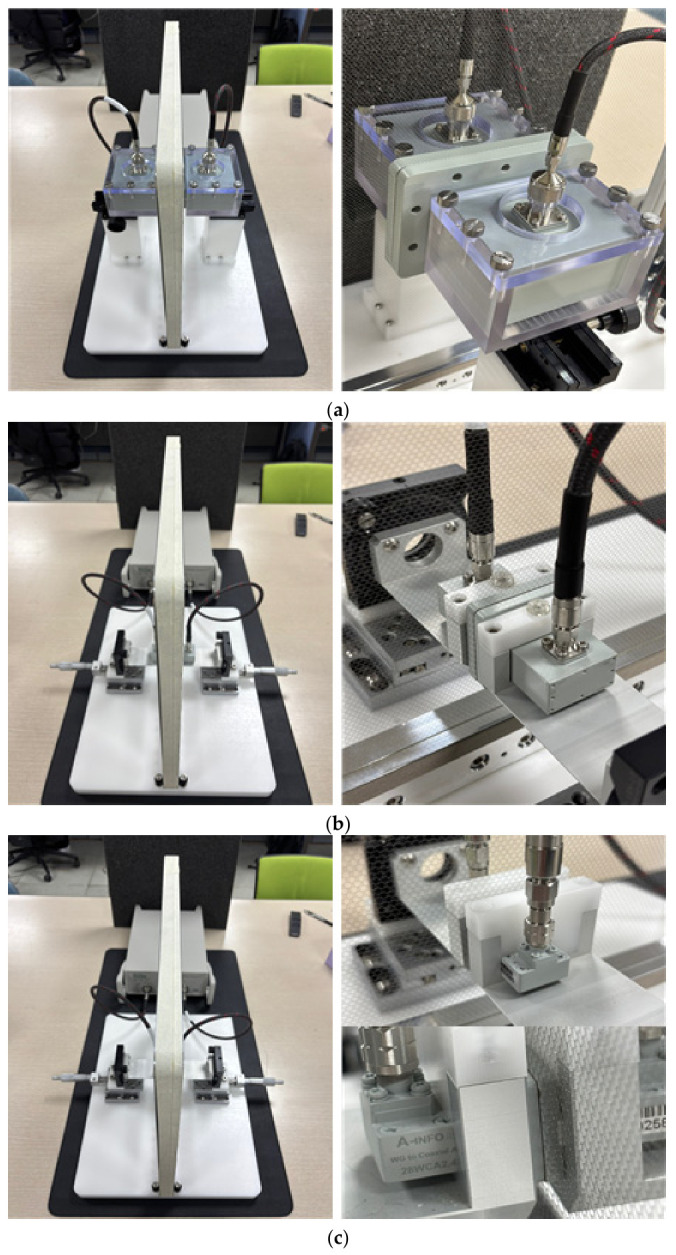
Waveguide-contact measurement fixture and setup: (**a**) WR-340 setup, (**b**) WR-90 setup, and (**c**) WR-28 setup.

Measurements were performed using a Copper Mountain Technologies S5243 vector network analyzer (VNA) to measure the single-port reflection characteristic S_11_. A dedicated fixture designed for each waveguide standard was used to maintain consistent contact conditions between the waveguide and specimen, and the fixture structure and mounting state are shown in Figure 2. Prior to measurement, Thru–Reflect–Line (TRL) calibration was applied to move the reference plane to the specimen contact surface, thereby correcting systematic errors attributable to the waveguide and fixture.

The reflection coefficient $S_{11}^{WG}$ measured under waveguide-contact conditions includes the boundary conditions of the waveguide’s intrinsic mode and is therefore difficult to compare directly with free-space reflection characteristics. Accordingly, the equivalent input impedance of the specimen was first derived from the measured reflection coefficient, then converted to the reflection coefficient by applying it to the free-space boundary conditions:

(9) $$Z_{Spec}=Z_{WG}\frac{1+S_{11}^{WG}}{1-S_{11}^{WG}}$$

where $Z_{WG}$ is the characteristic impedance of the waveguide used. Subsequently, assuming normal incidence free-space conditions, the measured free-space reflection coefficient $\Gamma_{meas}$ was calculated as:

(10) $$\Gamma_{meas}=\frac{Z_{Spec}-\eta_{0}}{Z_{Spec}+\eta_{0}}$$

### 3.2. Measured Reflection Coefficients of Mesh Specimens

This section presents the free-space reflection coefficient $\Gamma_{meas}(f)$ derived by the procedure in Section 3.1 for the three mesh specimens. Each specimen was measured across all three waveguide standards, and the free-space reflection coefficient was converted from $S_{11}^{WG}(f)$ via the equivalent impedance $Z_{Spec}(f)$.

Taking into account the directionality of the woven mesh structure, reflection coefficients were measured in two orthogonal polarization directions (H, V) with respect to the waveguide aperture. Here, the two polarizations are defined based on the electric field direction in the fundamental mode of the waveguide.

**Figure 3:**
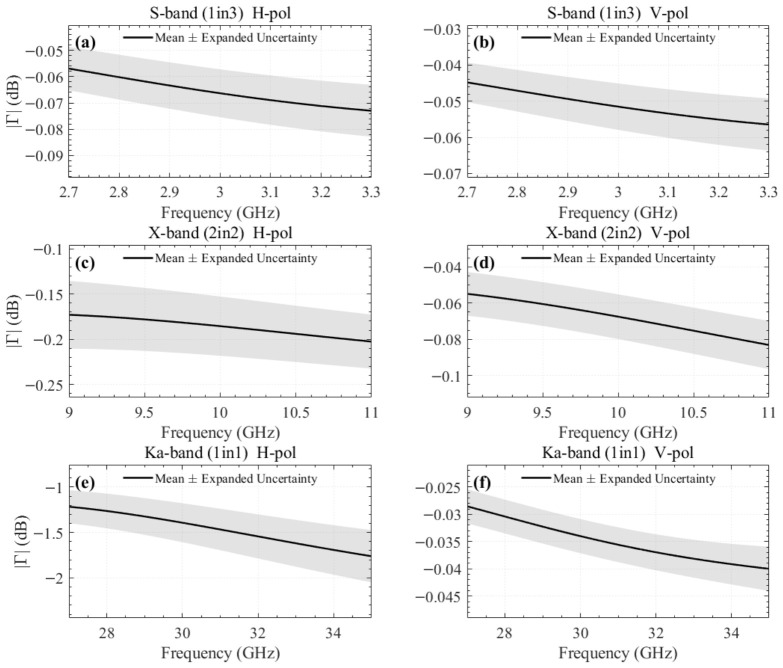
Measured free-space reflection coefficients of the mesh specimens (mean ± expanded uncertainty, 9 trials): (**a**) S-band 1in3 Atlas, H-pol; (**b**) S-band 1in3 Atlas, V-pol; (**c**) X-band 2in2 Atlas, H-pol; (**d**) X-band 2in2 Atlas, V-pol; (**e**) Ka-band 1in1 Atlas, H-pol; and (**f**) Ka-band 1in1 Atlas, V-pol. Shaded bands represent the expanded uncertainty at a 95% confidence level.

Figure 3 shows the frequency response of $\Gamma_{meas}(f)$ derived for the three specimens. The shaded bands indicate the expanded uncertainty (95% confidence level) evaluated from nine repeated trials per configuration, following the GUM [27]. The measurement data were obtained by converting the waveguide scattering coefficients to the time domain, applying a 1 ns time gate centered at the reflected signal position (0 ns) to remove multiple reflections and background noise, and then inverse-transforming back to the frequency domain. This window width is sufficient to capture the direct reflection from the specimen while suppressing measurement noise, given that the short waveguide section between the calibration reference plane and the specimen surface produces negligible internal multiple reflections.

## 4. Verification of Mesh Modeling

The applicability of the model is verified by comparing the Casey surface impedance model from Section 2 with the measurement results from Section 3. The Astrakhan PEC wire-grid model [14,15,16] is included for comparison under two conditions: with the physical wire radius (r = 15 μm, no fitting) and with the fitted effective wire radius. Since actual mesh fabrics have structural differences from simple cylindrical wire arrays, the wire radius *r_eff_* is treated as an equivalent parameter, and the optimal value reproducing the measured reflection coefficient is derived. The lattice spacings $a_{H}$ and $a_{V}$ use values derived from the specimen geometry, and the electrical conductivity is fixed at the material property of gold, σ = 45.2 MS/m. *r_eff_* is defined according to Equation (8) as the value minimizing the error between $\Gamma_{meas}(f)$ and $\Gamma (f,\;r_{eff})$ across each measured frequency band, and is estimated as a single value for each band. Optimization was performed separately for two orthogonal polarizations (H, V) in consideration of the directionality of the woven mesh fabric.

Table 2 summarizes the effective wire radius *r_eff_* estimated for three frequency bands and two directions. The expanded uncertainty U of *r_eff_* was evaluated by fitting *r_eff_* independently to each of the nine repeated measurement trials; the Type A standard uncertainty was obtained from the standard deviation of the fitted values, and the expanded uncertainty was reported at a 95% confidence level using the coverage factor from the t-distribution, following the GUM [27].

Since the three specimen groups in Table 2 correspond to physically distinct fabrics with different knit densities (Figure 1), the differences in *r_eff_* are expected to largely reflect fabric structure. Each estimated *r_eff_* is considered valid for characterizing the target specimen within and near the measured band. For the S-band 1in3 Atlas specimen, *r_eff_* was estimated at 17.8 μm in the H-direction and 10.1 μm in the V-direction. In the X-band, *r_eff_* values of 38.7 μm in the H-direction and 21.8 μm in the V-direction were obtained. In the Ka-band, values of 44.5 μm in the H-direction and 44.0 μm in the V-direction were obtained. Overall, *r_eff_* is interpreted not as the physical thickness of the yarn, but as a specimen-intrinsic parameter that equivalently incorporates the combined effects of multilayer structure, contact resistance, surface roughness, and yarn bundling.

**Figure 4:**
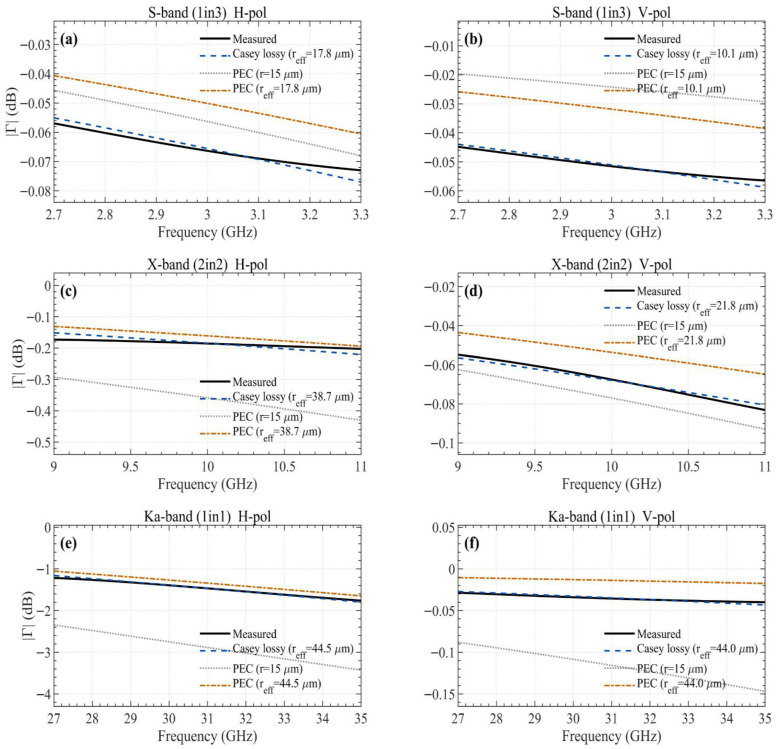
Comparison of measured reflection coefficients with Casey lossy model, Astrakhan PEC (r = 15 μm), and Astrakhan PEC with fitted effective wire radius: (**a**) S-band 1in3 Atlas, H-pol; (**b**) S-band 1in3 Atlas, V-pol; (**c**) X-band 2in2 Atlas, H-pol; (**d**) X-band 2in2 Atlas, V-pol; (**e**) Ka-band 1in1 Atlas, H-pol; and (**f**) Ka-band 1in1 Atlas, V-pol.

Figure 4 shows the comparison between measured values, the Casey lossy model, and the Astrakhan PEC model under two conditions. The Casey lossy model with fitted effective wire radius shows excellent agreement with the measured reflection coefficients, with native-band RMSE ranging from 0.001 dB to 0.021 dB (Table 3). The Astrakhan PEC model with the physical wire radius (r = 15 μm) agrees well at S-band but progressively diverges at higher frequencies. When the same fitted effective wire radius is applied to the Astrakhan PEC model, the resulting curves closely follow those of the Casey lossy model across all bands, indicating that the dominant factor in reproducing the measured reflection coefficients is the effective wire radius parameter rather than the choice between PEC and lossy formulations. Further discussion is provided in Section 5.3.

Even for the same specimen, directional *r_eff_* values differed due to the anisotropy of the mesh weave structure. The *r_eff_* in the H-direction was approximately 1.76× that of the V-direction in the S-band, approximately 1.78× in the X-band, and approximately 1.01× in the Ka-band. This anisotropy implies that polarization dependence must be considered in mesh reflector antenna design.

## 5. Discussion

### 5.1. Physical Interpretation of the Effective Wire Radius

The yarn used in all three specimens is gold-coated molybdenum wire with a physical diameter of 30 μm (radius 15 μm). The fitted effective wire radius values range from 10.1 to 44.5 μm across all configurations, deviating from the physical radius systematically. Specimens with higher knit density, as visible in the optical images in Figure 1, tend to exhibit larger effective wire radius values. The 1in1 Atlas (Ka-band specimen) has the densest construction, while the 1in3 Atlas (S-band specimen) has the most open structure. Since denser knit patterns involve greater wire overlap and inter-wire contact area, the effective electromagnetic cross-section increases, which is captured by a larger effective wire radius. The anisotropy in the effective wire radius between H-polarization and V-polarization reflects the structural asymmetry of the Atlas knit pattern, where the wale and course directions have inherently different wire configurations. The effective wire radius should therefore be interpreted not as a physical dimension but as a specimen-intrinsic equivalent parameter that incorporates the combined effects of multilayer yarn structure, inter-wire contact resistance, surface roughness, and yarn bundling.

The electrical conductivity is fixed at the bulk gold value (45.2 MS/m) in this study. The actual conductivity of the gold coating may deviate from the bulk value due to factors such as coating thickness, surface roughness, and deposition conditions; however, incorporating finite conductivity through the Casey formulation represents a significant improvement over the PEC assumption adopted in earlier studies [14,15,16], which neglects conductive loss entirely. It should be noted that *r_eff_* and σ are coupled parameters in the model: if the actual coating conductivity differs from the assumed bulk gold value, the fitted *r_eff_* would shift to compensate for the discrepancy in resistive loss. Therefore, the *r_eff_* values reported in this study are valid under the bulk gold conductivity assumption and should be re-estimated if a measured coating conductivity becomes available.

### 5.2. Validity Range and a/λ Analysis

The Casey surface impedance model is a dominant-mode approximation. As the lattice-period-to-wavelength ratio a/λ increases, higher-order Floquet modes become significant, and accuracy degrades. Following Yatsenko et al. [21], this threshold is approximately a/λ ≈ 0.3. All inner-spacing values in this study satisfy a/λ < 0.3 up to 35 GHz (the upper edge of WR-28), supporting the validity of the single-mode assumption for the tested configurations.

### 5.3. Comparison with Existing Models

Figure 4 includes the Astrakhan PEC wire-grid model [14,15,16] under two conditions: with the physical wire radius (r = 15 μm) and with the fitted effective wire radius. With the physical wire radius, the Astrakhan PEC model and the Casey lossy model produce closely matched curves at S-band, where the lattice period is small relative to the wavelength. However, as frequency increases, the PEC model with the physical radius progressively diverges: in the X-band, it systematically underestimates the reflectivity, and in the Ka-band H-polarization, the discrepancy reaches approximately 2 dB. This divergence occurs because the physical wire radius (15 μm) does not account for the effective electromagnetic cross-section enhancement caused by the complex knit structure. When the same fitted effective wire radius is applied to the Astrakhan PEC model, the resulting curves closely follow those of the Casey lossy model across all bands, indicating that the dominant factor in reproducing the measured reflection coefficients is the effective wire radius rather than the choice between PEC and lossy formulations. The difference between the two formulations is secondary. Rather than claiming superiority of one formulation over another, this paper presents a procedure for extracting the effective wire radius from waveguide-contact measurements.

## 6. Conclusions

In mesh reflector antenna reflective surface modeling, while studies applying scaling factors to the strip aperture model have been reported [24,25], cases of applying a scaling procedure to wire grid models incorporating finite electrical conductivity for reflection coefficient characterization are difficult to find. This paper addressed this gap by presenting a modeling method that combines a per-band effective wire radius *r_eff_* estimation procedure with the Casey surface impedance model. The lattice spacing was fixed from the specimen geometry, the electrical conductivity was set to the value for gold (σ = 45.2 MS/m), and *r_eff_* was optimized across each measured frequency band, thereby constructing a parameter determination procedure that does not depend on manufacturing specifications or cross-sectional measurements.

For validation, waveguide-contact measurements were performed on three Atlas-series mesh specimens fabricated with gold-coated molybdenum wire, measuring each specimen across all three waveguide standards with nine repeated trials (162 total runs). The estimated *r_eff_* ranged from 10.1 to 44.5 μm depending on band and direction, and as confirmed in the comparison results in Figure 4, excellent agreement between measured and model-predicted values was observed across all bands. This supports the conclusion that scaling by a single effective wire radius parameter is an appropriate approach for reproducing the reflective characteristics of complex woven/knitted structures. Furthermore, it was confirmed that even for the same specimen, directional *r_eff_* values differ by up to 1.78× due to the anisotropy of the weave structure. While the present approach is semi-empirical and the effective wire radius must be determined from measurements for each specimen configuration, the results demonstrate that this single-parameter calibration is sufficient to reproduce the measured reflection characteristics with high accuracy across all tested bands.

The modeling procedure presented in this study can be used as input data for incorporating reflective surface properties in numerical simulations of mesh reflector antennas, and can support more reliable estimates of reflective surface loss at target frequency bands and link budget calculations. Future work will extend the approach to oblique incidence, examine the frequency dependence of the effective wire radius, and incorporate higher-order impedance boundary conditions to improve accuracy at frequencies where a/λ approaches the validity limit. Additional directions include investigating the sensitivity of *r_eff_* to the assumed electrical conductivity and pursuing cross-validation with free-space or alternative measurement methods. In addition, since the metal mesh in actual deployable antennas is subjected to mechanical tension to maintain the reflector shape, the resulting changes in local knit density and inter-wire contact conditions may affect the effective wire radius; investigating the influence of applied tension on *r_eff_* constitutes an important direction for future work. Among these directions, establishing a quantitative relationship between *r_eff_* and the knit pattern parameters, such as the needle pattern type and the number of wire crossings per unit cell, would be a key step toward reducing the empirical dependence of the proposed method and enabling a priori parameter estimation for new mesh configurations without requiring additional measurements.

## Figures and Tables

**Table 1:** Waveguide standards and native measurement bands. All specimens were measured across all three waveguides.

Specimen	Measurement Frequency	Waveguide Used
S-band 1in3 Atlas	2.7–3.3 GHz	WR-340
X-band 2in2 Atlas	9–11 GHz	WR-90
Ka-band 1in1 Atlas	27–33 GHz	WR-28

**Table 2:** Estimated effective wire radius and expanded uncertainty by frequency band.

Band	Pol.	Period [μm]	*r_eff_* [μm]	U [μm]
S-Band	H	$a_{V}$ = 1900	17.8	±1.53
S-Band	V	$a_{H}$ = 1389	10.1	±1.39
X-Band	H	$a_{V}$ = 1565	38.7	±5.22
X-Band	V	$a_{H}$ = 892	21.8	±1.91
Ka-Band	H	$a_{V}$ = 1643	44.5	±7.28
Ka-Band	V	$a_{H}$ = 485	44.0	±2.48

**Table 3:** Native-band fitting RMSE between the mean measurement and the Casey lossy model.

Specimen	Pol.	*r_eff_* [μm]	RMSE [dB]
S (1in3)	H	17.8	0.0017
S (1in3)	V	10.1	0.0009
X (2in2)	H	38.7	0.0112
X (2in2)	V	21.8	0.0014
Ka (1in1)	H	44.5	0.0206
Ka (1in1)	V	44.0	0.0014

## Data Availability

The data presented in this study are available on request from the corresponding author.
